# MicroRNA-128b mediates lipopolysaccharide-induced apoptosis via reactive oxygen species in human pulmonary microvascular endothelial cells

**DOI:** 10.1016/j.clinsp.2022.100020

**Published:** 2022-03-16

**Authors:** Guangwen Long, Xiulin Yang, Chunling Ji, Yukang Dong

**Affiliations:** Emergency Department of Internal Medicine, Guizhou Provincial People's Hospital, Guiyang, China

**Keywords:** miR-128b, PRKD1, Apoptosis, Reactive oxygen species, Human pulmonary microvascular endothelial cells

## Abstract

**Objectives:**

This study aimed to explore the effects of miR-128b in the regulation of Lipopolysaccharide (LPS) induced apoptosis.

**Methods:**

Human Pulmonary Microvascular Endothelial Cells (HPMECs) were transfected with an miR-128b inhibitor and stimulated with LPS for 24 h. FCM was performed to detect apoptosis and Reactive Oxygen Species (ROS) production. In addition, miRNA and caspase-3 expression levels were determined using real-time quantitative polymerase chain reaction and western blotting.

**Results:**

LPS significantly induced apoptosis and ROS production and upregulated miR-128b and caspase-3 expressions in HPMECs. However, LPS-induced effects were suppressed when an miR-128b inhibitor was used. Preincubation with NAC decreased the LPS-induced apoptosis of HPMECs.

**Conclusions:**

These effects were mediated by miR-128b via the caspase-3 pathway.

## Highlights

• We observed that miR-128b regulated LPS-induced apoptosis of HPMECs and identified its mechanism.
• LPS induced apoptosis and ROS production and upregulated miR-128b and caspase-3 expressions in HPMECs.
• miR-128b has a potential therapeutic target for the treatment of ARDS.

## Introduction

Acute Respiratory Distress Syndrome (ARDS), characterized by hypoxemia and overwhelming pulmonary inflammation, is widely recognized as a group of lung diseases associated with high morbidity and mortality. At present, the treatment and early diagnosis of ARDS are limited. During ARDS, severe inflammatory responses induce apoptosis as well as the release of fibrotic agents, which contribute to the pathogenesis of the lungs.^1^ The main feature of infectious and noninfectious lung injury is lung epithelial cell damage. It plays a vital role in the pathogenesis of vascular leaking and severe inflammation involved in ARDS.^2^ However, it remains unclear how lung inflammation initiates and spreads during the development of lung damage, particularly through certain infectious stimuli.

As small non-protein-coding RNAs, microRNAs (miRNAs) serve as negative regulators of gene expression.^3^ Recently, miRNAs have been reported to be involved in various biological and pathological processes, including ARDS.^4^^,^^5^ miR-128b is a rarely studied miRNA that is a potent glucocorticoid sensitizer in acute lymphocytic leukemia cells^6^ miR-128 inhibits cell proliferation, tumor growth, and angiogenesis in gliomas.^7^ However, whether miR-128 is involved in the pathogenesis of ARDS remains to be further explored.

In this study, the authors investigated the role of miR-128b in the regulation of Lipopolysaccharide (LPS) induced Human Pulmonary Microvascular Endothelial Cell (HPMEC) apoptosis. Further, the authors investigated the influence of LPS on the regulation of apoptosis and Reactive Oxygen Species (ROS) production. In addition, to identify the potential role of miR-128b as a mediator of the effects of LPS on apoptosis via ROS production, the authors transfected HPMECs with an miR-128b inhibitor. Finally, the possible role of the caspase-3 pathway in LPS-mediated effects was analyzed.

## Materials and methods

### Collection of human blood samples

This study was approved by the Research Ethics Committee of the Guizhou Provincial 'People's Hospital. The authors collected plasma from 10 severe pneumonia patients, and they have fulfilled ARDS criteria (the Berlin Definition). After blood collection, plasma was prepared by centrifuging the blood samples at 1000 × g for 10 min. The Trizol reagent was added, and the preparations were stored at -80 °C until further use.

### Cell culture and treatment

HPMECs were maintained in 'Dulbecco's Modified Eagle Medium (DMEM) (Hyclone) supplemented with 10% Fetal Bovine Serum (FBS) (Gibco) and 1% PS (Beyotime Technology). For some experiments, HPMECs were treated with DMEM supplemented with 2% FBS and 1 µg/mL LPS (Sigma-Aldrich) for 24 h. For other experiments, HPMECs were pretreated with PRKD1 inhibitor (R&D System) for 2 h. Cells treated with PBS served as the control group.

### miRNA transfection

The authors used Lipofectamine 2000 (Invitrogen) to transfect miR-128b NC and inhibitor into the cells and then harvested the cells 24 h after transfection for subsequent RNA and protein extraction. The sequences of the miRNAs are as follows: miRNA-128-b inhibitor, 5′-AAAGAGACCGGUUCACUGUGA-3′ and inhibitor NC, 5′-CAGUACUUUUGUGUAGUACAA-3′ (Guangzhou RiboBio).

### Real-time quantitative polymerase chain reaction (qRT-PCR)

Total RNA was isolated using the Trizol reagent (Vazyme Biotech). cDNA was synthesized using the miRNA 1st Strand cDNA Synthesis Kit (Vazyme Biotech). RT-PCR was performed using the SYBR Premix (Vazyme Biotech) on an Applied Biosystems 7500 Real-Time PCR system (Applied Biosystems). Glyceraldehyde 3-Phosphate Dehydrogenase (GAPDH) was used as an internal control. The ABI software was used to determine Ct values, and gene expression levels were calculated using the 2^−ΔΔCt^ method. The primers for miR-128b, AK2, and PRKD1 were synthesized by Vazyme Biotech. The primer sequences are as follows: miR-128b, 5′-UCACAGUGAACCGGUCUCUUU-3′; miR-128b inhibitor, 5′-AAAGAGACCGGUUCACUGUGA-3′; inhibitor NC, 5′-CAGUACUUUUGUGUAGUACAA-3′; PRKD1, 5′-TTCTCCCACCTCAGGTCATC-3′ and 5′-TGCCAGAGCACATAACGAAG-3′; and AK2, 5′-GCAGAACCCGAGTATCCTAAAGG-3′ and 5′-TTCCCAGCATCCATAGTTGCC-3′ (Guangzhou RiboBio).

### Western blotting

Cell lysates were harvested, and protein concentrations were measured using the BCA Kit (Beyotime Technology). The monoclonal anti-cleaved caspase-3 antibody (Cell Signaling Technology), anti-AK2 antibody (Abcam), anti-PRKD1 (Solarbio), and anti-GAPDH antibody (Proteintech) were used as the primary antibodies. Goat anti-mouse IgG (Proteintech) was used as the secondary antibody.

### Flow cytometric analysis

For apoptosis analysis, HPMECs were washed with binding buffer and stained using the Annexin V/PI Apoptosis Kit (Miltenyi Biotec) according to the 'manufacturer's instructions. After staining at room temperature for 15 min, the cells were analyzed using an FCM (BD Biosciences).

For intracellular ROS detection, 2.7-Dichlorofluorescein Diacetate (DCFHDA, Sigma-Aldrich) was used. After stimulating the cells with LPS for 24 h, HPMECs were incubated with DCFHDA in DMEM at 37 °C for 30 min. After washing twice with PBS, the mean fluorescence intensity was measured using an FCM (BD Biosciences) at an excitation wavelength of 488 nm and an emission wavelength of 538 nm.

### Statistical analysis

Statistical analysis was performed using the 'Wilcoxon's signed-rank test or one-way analysis of variance. Data are represented as mean ± SEM. A *p*-value of < 0.05 was considered statistically significant. The Graphpad 7.0 software was used for statistical analysis.

## Results

### LPS induced HPMEC apoptosis and ROS production

**Figure 1.**
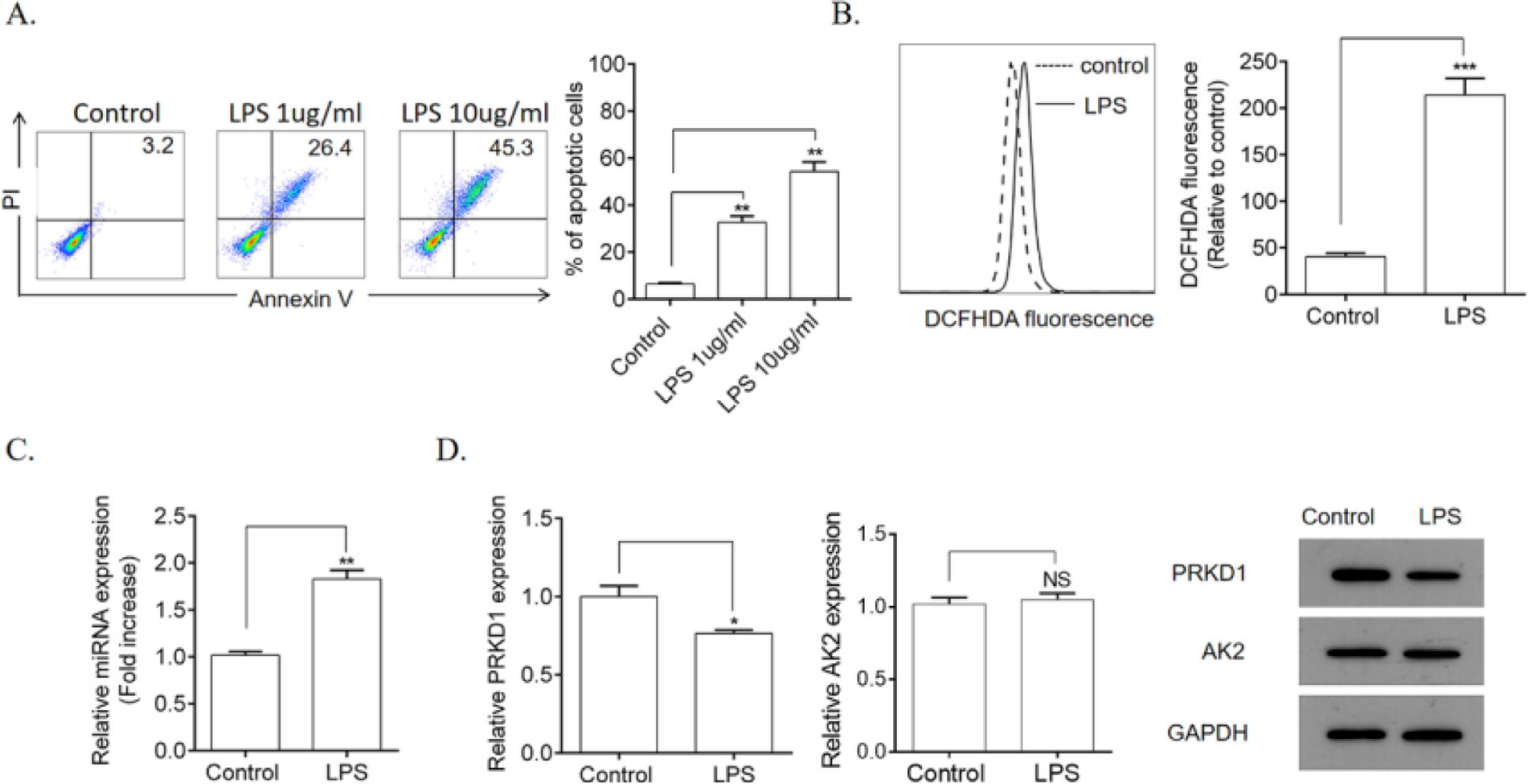
Detection of Human Pulmonary Microvascular Endothelial Cell (HPMEC) apoptosis via FCM analysis. HPMECs were treated with Lipopolysaccharide (LPS) (1 and 10 μg/mL) for 24 h. Apoptosis was detected via FCM analysis. Apoptotic cells were expressed as annexin V- and PI-positive cells. (A) Reactive Oxygen Species (ROS) production was measured using 2.7-Dichlorofluorescein Diacetate (DCFHDA) (10 µM) via FCM analysis (*n* = 4). (C, D) Expression levels of miRNA, PRKD1, and AK2 were detected using real-time quantitative Polymerase Chain Reaction (qRT-PCR) and western blotting. HPMECs were evaluated after treatment with LPS (1 μg/mL) or PBS for 24 h. Gene and protein expression levels were normalized to those of the control group (*n* = 4). Data are represented as mean ± SEM. *p < 0.05, **p < 0.01, ***p < 0.001.

First, the authors explored the influence of LPS on HPMEC apoptosis and ROS production. Compared with the control group, stimulation with LPS (1 and 10 µg/mL) remarkably induced apoptosis (Fig. 1A) as well as significantly increased ROS production (Fig. 1B) in HPMECs. Furthermore, it remarkably upregulated the gene expression levels of miR-128b (Fig. 1C). Using TargetSCAN (http://www.targetscan.org), the authors identified the transcriptional repressors PRKD1 and AK2 as the putative targets of miR-128b. However, the gene and protein expression levels of PRKD1 were decreased by LPS, whereas those of AK2 remained unchanged (Fig. 1D).

### miR-128b inhibitor blocked the effect of LPS on miR-128b gene expression

**Figure 2.**
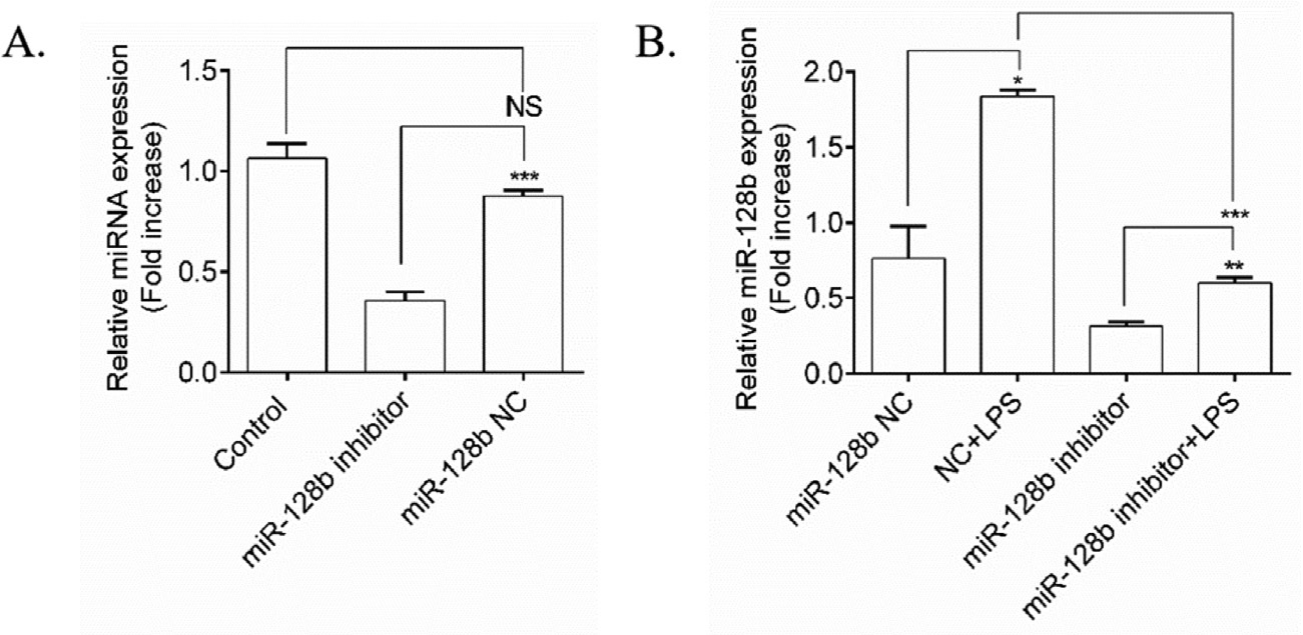
(A, B) miRNA expression using real-time quantitative Polymerase Chain Reaction (qRT-PCR). Human Pulmonary Microvascular Endothelial Cells (HPMECs) were evaluated after 24 h of transfection with an miR-128b inhibitor (50 nM) or NC (10 nM) treated with Lipopolysaccharide (LPS) (1 μg/mL). The gene and protein expression levels were normalized to those of the control group or NC (*n* = 4). Data are represented as mean ± SEM. *p < 0.05, **p < 0.01, ***p < 0.001.

To determine whether the expression levels of miR-128b were induced by LPS, an miR-128b inhibitor was transfected into HPMECs (Fig. 2A). qRT-PCR revealed that the miRNA inhibitor remarkably downregulated the gene expression levels of miR-128b compared with the control group and NC. After transfection with the miR-128b inhibitor, the gene expression levels of miR-128b were upregulated in LPS-stimulated HPMECs (Fig. 2B).

### miR-128b inhibitor prevented the LPS-Induced Apoptosis of HPMEC via PRKD1

**Figure 3.**
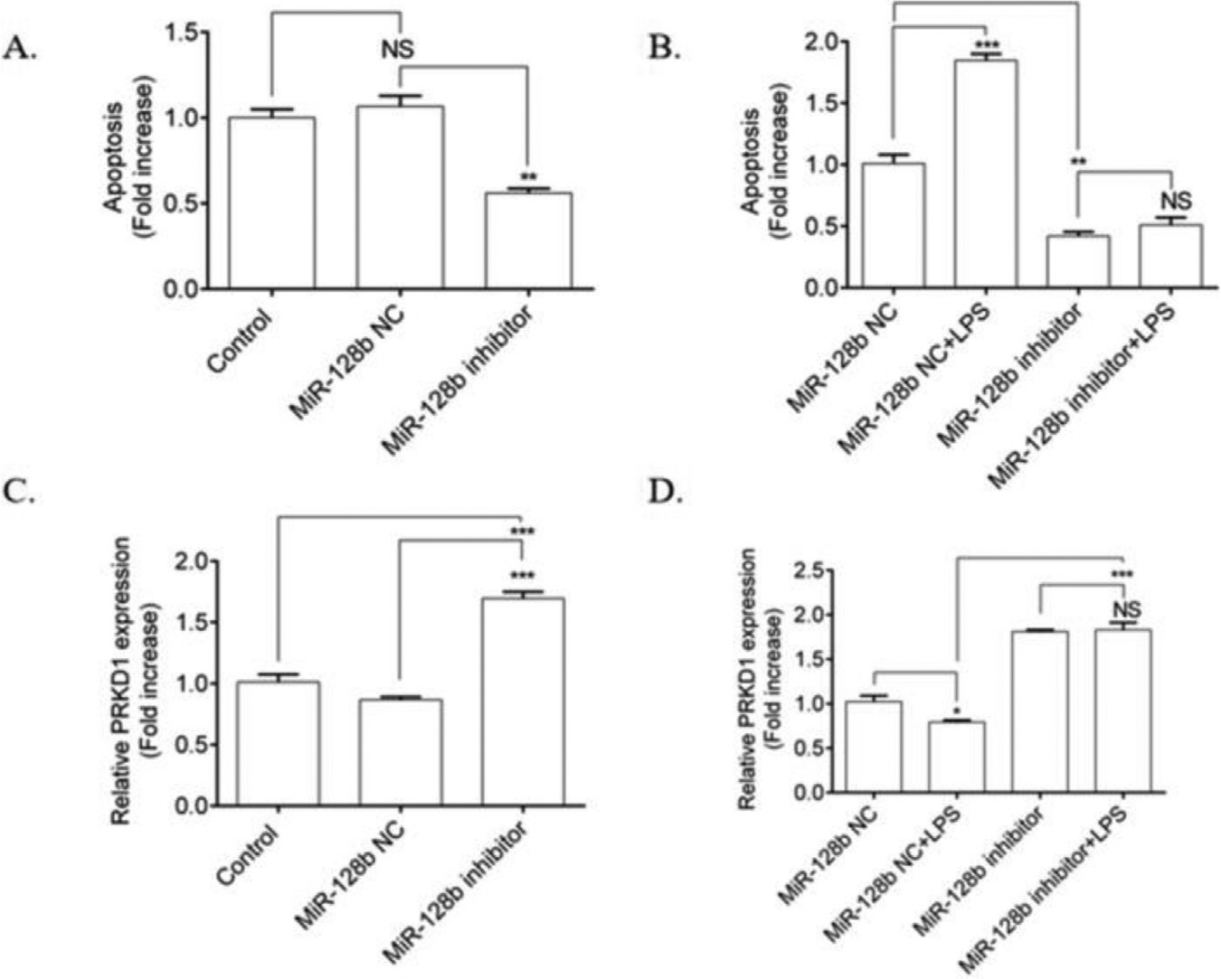
(A-) Detection of human pulmonary microvascular endothelial cell (HPMEC) apoptosis via FCM analysis. HPMECs were treated with Lipopolysaccharide (LPS) (1 μg/mL) for 24 h. Apoptosis was analyzed via FCM analysis. Apoptotic cells were defined as annexin V- and PI-positive cells. (C, D) Expression levels of PRKD1 determined using real-time quantitative Polymerase Chain Reaction (PCR). HPMECs were evaluated after 24 h of transfection with an miR-128b inhibitor (50 nM) or NC (10 nM) and after 24 h treatment stimulus with LPS (1 μg/mL) (*n* = 4). Data are represented as mean ± SEM. *p < 0.05, **p < 0.01, ***p < 0.001.

The authors investigated the effects of LPS on the apoptosis of HPMECs transfected with an miR-128b inhibitor. HPMECs transfected with the miR-128b inhibitor exhibited a significant decrease in apoptosis (Fig. 3A) and an upregulation in the expression of PRKD1 compared with the NC and control (Fig. 3C). LPS stimulation significantly increased the number of apoptotic cells and decreased the expression levels of PRKD1 in the cells transfected with NC. In contrast, after incubation with the miR-128b inhibitor, there was no influence of LPS on apoptosis and PRKD1 expression in LPS-treated HPMECs (Fig. 3B, D).

### miR-128b inhibitor regulated LPS-Induced ROS production

**Figure 4.**
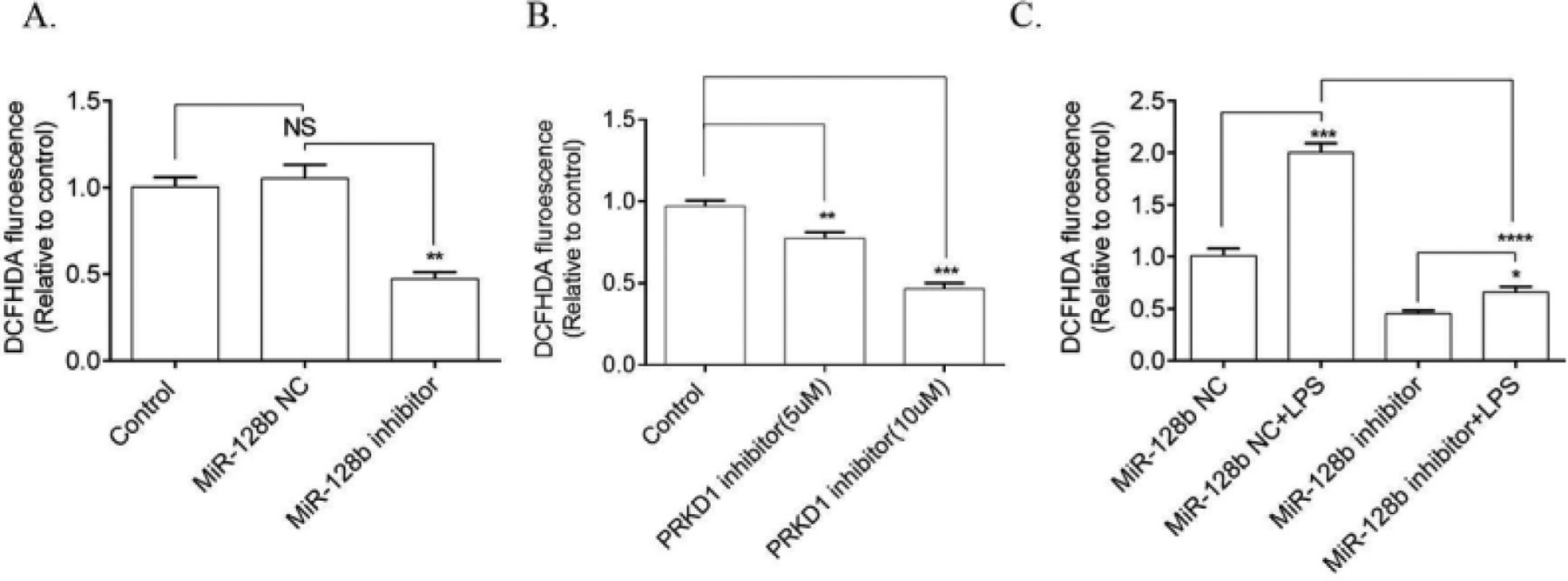
(C) Reactive Oxygen Species (ROS) production via FCM analysis using 2,7-Dichlorofluorescein Diacetate (DCFHDA). ROS production in Human Pulmonary Microvascular Endothelial Cells (HPMECs) was evaluated after 24h of transfection with an miR-128b inhibitor (50 nM), NC (10 nM), or CRT0066101 (5 μM, 10 μM) and after 24 h treatment with Lipopolysaccharide (LPS) (1 μg/mL) (*n* = 4). Data are represented as mean ± SEM. *p < 0.05, **p < 0.01, ***p < 0.001, ****p < 0.0001.

ROS production was significantly downregulated in HPMECs transfected with the miR-128b inhibitor compared with NC (Fig. 4A). Moreover, PRKD1 inhibitor CRT0066101 decreased the level of ROS (Fig. 4B). The effect of LPS on ROS production was significantly inhibited by the miR-128b inhibitor (Fig. 4C).

### LPS-Induced HPMEC apoptosis was mediated by ROS

**Figure 5:**
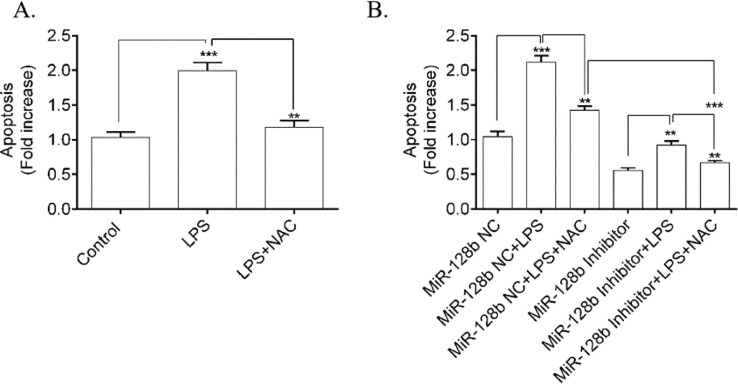
(A, B). Detection of human pulmonary microvascular endothelial cell (HPMEC) apoptosis via FCM analysis. Apoptosis was detected after 24h of transfection with an miR-128b inhibitor (50 nM) or NC (10 nM) and after 24 h treatment with Lipopolysaccharide (LPS) (1 μg/mL) or NAC (*n* = 4). Data are represented as mean ± SEM. *p < 0.05, **p < 0.01, ***p < 0.001.

To determine whether ROS was involved in LPS-induced HPMEC apoptosis, the authors used NAC (a ROS scavenger) to block the effect of ROS. FCM analysis showed a significant decrease in apoptosis (Fig. 5A) in HPMECs treated with NAC alone or when coincubated with LPS compared with the control. Furthermore, NAC blocked the apoptosis of HPMECs transfected with the miR-128b inhibitor (Fig. 5B).

### miR-128b decreased LPS-induced HPMEC apoptosis via the caspase-3 signaling pathway

**Figure 6:**
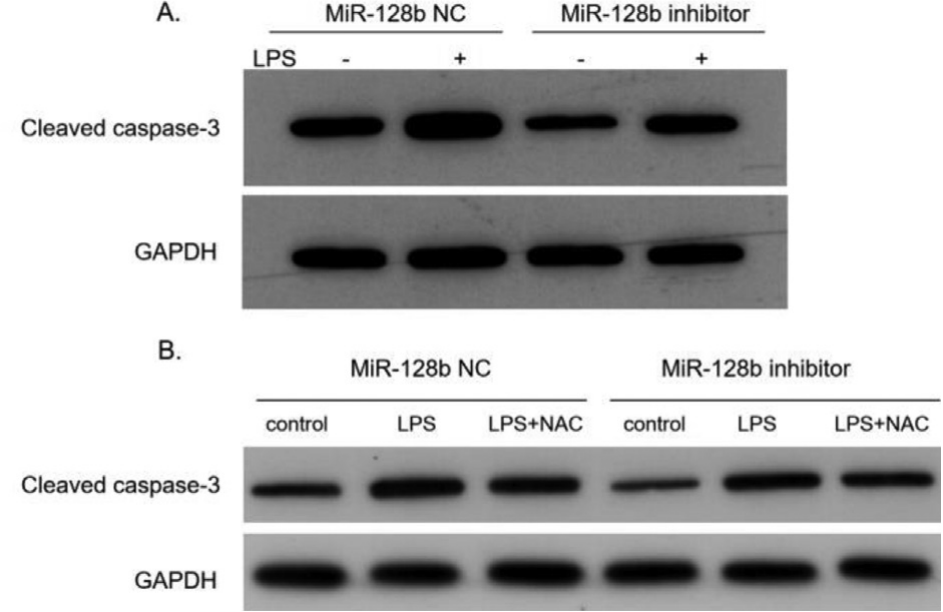
(A, B) Representative western blots of cleaved caspase-3 in Human Pulmonary microvascular Endothelial Cells (HPMECs) transfected with an miR-128b inhibitor and NC. HPMECs were treated with LPS (1 μg/mL) or NAC (10 μM) (*n* = 3).

After transfection with the miR-128b inhibitor, the expression levels of caspase-3 in HPMEC were detected via western blotting. The data indicated that the expression levels of caspase-3 were significantly decreased in HPMECs transfected with the miR-128b inhibitor than in control cells (Fig. 6A). After adding NAC, the expression levels of caspase-3 were downregulated (Fig. 6B).

### miR-128b showed a different expression in time-related variation in the peripheral blood of patients with ARDS

**Figure 7:**
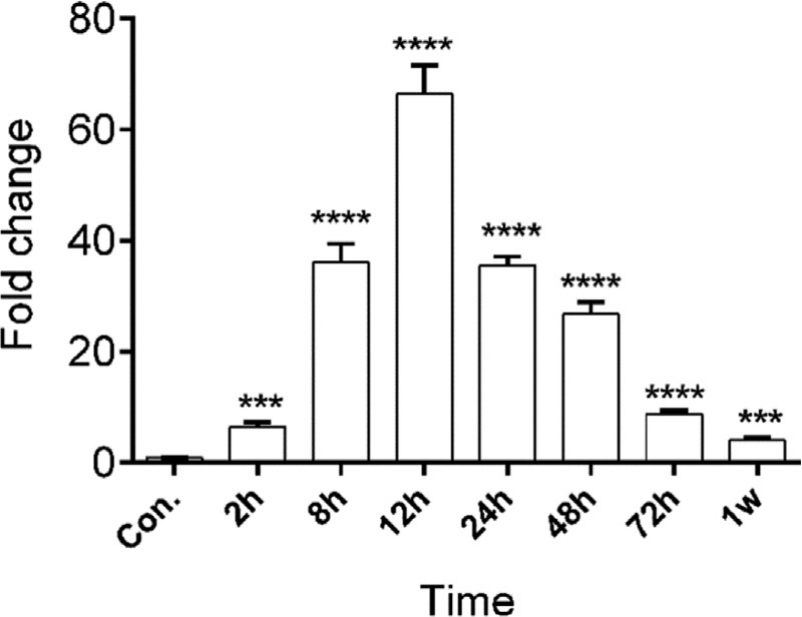
miR-128b expression levels in the peripheral blood samples of patients with Acute Respiratory Distress Syndrome (ARDS) (*n* = 10) and healthy controls (*n* = 10) at different hours after the onset of ARDS. ****p < 0.0001.

The authors studied the expression levels of miR128b in the plasma of 10 healthy people and 10 patients with ARDS (Table 1) at different times of onset. RT-PCR revealed that the expression levels of miR-128b were upregulated at 2, 8, 12, 24, 48, and 72 h after the onset of ARDS and that they were most obviously upregulated at 12 h and then returned to their normal levels after one week. These results suggest that miR-128b is closely associated with ARDS progress and might be a potential biomarker of ARDS (Fig. 7).

**Table 1:** Clinical characteristics of ARDS patients.

**Patient, n°**	**Age/Sex**	**Lung injury score**	**PaO_2_/FiO_2_ (mm Hg)**	**Coexisting conditions**
01	55/F	2.5	96.6	Lung cancer
02	65/M	2.7	89.7	Coronary heart disease
03	43/M	2.4	135.6	Trauma
04	58/F	2.1	121.8	Lung cancer
05	74/M	2.5	105.8	Hypertension
06	41/M	2.3	125.2	Trauma
07	55/F	2.6	132.1	Coronary heart disease
08	74/F	2.3	145.2	Lung cancer
09	69/M	2.4	100.7	Coronary heart disease
10	77/M	2.6	97.8	Chronic renal disease

## Discussion

ARDS is a severe inflammatory disease characterized by pulmonary epithelial and endothelial cell dysfunction, inflammatory cell infiltration, and apoptosis. Severe inflammatory cascades impair the regulation of the vascular endothelial barrier as well as its permeability.^1^^,^^8^ Therefore, it is important to explore the microenvironment of pulmonary cells to understand the development and management of ARDS. It has been reported that the balance between the survival and death of endothelial cells plays an important role in the prognosis of ARDS. Because endothelial cells are located at the interface between the circulating blood and surrounding tissues, they may come into contact with various environmental risk factors, including bacteria, hypoxia, hyperoxia, and oxidants.^9^, ^10^, ^11^ miRNAs are a growing family of small RNAs that function as post-transcriptional regulators of gene expression. Several reports have reported that miRNAs are involved in several diseases, including cardiac disorders^12^ and kidney diseases.^13^ Another study has identified few miRNAs as biomarkers and therapeutic targets of ARDS. It has been reported that miRNAs are involved in macrophage polarization, thereby regulating ARDS pathogenesis.^14^ In the present study, the authors found that miR-128b regulated LPS-induced apoptosis of HPMECs and identified its mechanism.

Apoptosis plays an important role in the homeostasis and pathogenesis of many diseases.^15^^,^^16^ It eliminates unwanted cells and may be beneficial for physiological processes. However, excessive or inappropriate apoptosis can result in human diseases. The vascular endothelium is a crucial intravascular compartment that serves as a barrier and plays an essential role in several physiological and pathological processes.^17^ Endothelial cells are involved in the regulation of blood flow, wound healing, coagulation, leukocyte trafficking, and angiogenesis.^18^

Apoptosis aggravates lung injury. However, the apoptosis of pulmonary vascular endothelial cells plays an important role in the occurrence of diseases and other physiological processes. In addition, it may initiate or contribute toward the progression of lung diseases. LPS has been detected in the blood of patients with gram-negative septicemia. Furthermore, circulating LPS levels can predict the development of multiorgan failure in patients with ARDS.^19^ In this study, LPS was used to induce the apoptosis of HPMECs. Moreover, the authors showed that miR-128b regulated the progress of LPS-induced HPMEC apoptosis via PRKD1 expression. After transfection with miR-128b, apoptosis was inhibited. The present study's results suggest that miR-128b has a proapoptotic effect on HPMECs.

ROS is one of the main endogenous oxidants in cell respiration and the 'host's response to infection.^20^ It plays a pivotal role in redox signaling pathways. ROS principally originates from mitochondrial production. H_2_O_2_, a form of ROS, has been used in redox biology to activate biological processes by activating signaling pathways due to its non-radioactive nature and transmembrane migration ability.^21^ Furthermore, some miRNAs have been reported as oxidative stress-related miRNAs.^22^^,^^23^ miR-154-5p was reported to increase Angiotensin II-induced cardiac hypertrophy.^24^ miRNA-34a and miRNA-181a mediated oxidative stress in visfatin-induced cell apoptosis.^25^ In the present study, the authors proved that LPS induced the apoptosis of HPMECs via ROS production and that miR-128b was involved in this progress using its inhibitor. Furthermore, the authors found that the caspase-3 signaling pathway contributed to this progress. The authors collected blood samples from patients with ARDS and healthy controls and found that the expression levels of miR-128b were upregulated within one week after ARDS onset. This result may provide a potential early predictor for ARDS.

In summary, the present study's results may provide new information regarding the molecular mechanism of LPS-induced apoptosis of HPMECs, which may be regulated by miR-128b and the caspase-3 signaling pathway. These findings highlight the importance of miR-128b as a potential therapeutic target for the treatment of ARDS.

## CRediT authorship contribution statement

**Guangwen Long:** Visualization, Writing – review & editing. **Xiulin Yang:** Visualization, Formal analysis, Investigation, Writing – review & editing. **Chunling Ji:** Formal analysis, Investigation. **Yukang Dong:** Formal analysis, Investigation.

## Conflicts of interest

The authors declare no conflicts of interest.
